# Supplementary material for: Lack of asmt1 or asmt2 Yields Different Phenotypes and Malformations in Larvae to Adult Zebrafish
Source: Int J Mol Sci. 2025 Apr 21;26(8):3912. doi: 10.3390/ijms26083912 (PMC12027777; doi:10.3390/ijms26083912)
Supplement: Supplementary file 1 [file ijms-26-03912-s001.zip › ijms-3570624-supplementary.pdf]

Table S1. sgRNA, forward and reverse primers sequences.

Type	Sequence
sgRNA	TAATACGACTCACTATAGGGCACCAGTGTGGACGGGAGTTTTAGAGCTAGAA
asmt1 primer forward	CTGTGAGCTGGGTGTGTTTG
asmt1 primer reverse	TACACACACACACACCGACG
asmt2 primer forward	GTCATTGACTATAGCAGAAAGGACA
asmt2 primer reverse	AGCAGGACCAGCCTATGACTT

Table S2. Malformations analysis in 5 dpf zebrafish. Post-hoc test: Tukey multiple comparison of the means

Groups	P value			
	Normal	Edema	Tail abnormalities	Yolk abnormalities
WT vs asmt1$^{+/-}$	ns	ns	ns	ns
WT vs asmt$^{-/-}$	$P < 0.05$	ns	ns	ns
WT vs asmt2$^{+/-}$	ns	ns	ns	ns
WT vs asmt2$^{-/-}$	ns	ns	ns	ns
asmt1$^{+/-}$ vs asmt1$^{-/-}$	ns	ns	ns	ns
asmt1$^{+/-}$ vs asmt2$^{+/-}$	ns	ns	ns	ns
asmt1$^{+/-}$ vs asmt2$^{-/-}$	ns	ns	ns	ns
asmt1$^{-/-}$ vs asmt2$^{+/-}$	$P < 0.01$	ns	ns	ns
asmt1$^{-/-}$ vs asmt2$^{-/-}$	ns	ns	ns	ns
asmt2$^{+/-}$ vs asmt2$^{-/-}$	ns	ns	ns	ns

Table S3. Malformations analysis in 2-year-old zebrafish. Post-hoc test: Tukey multiple comparison of the means

**Groups**	**P value**		
	Normal	Scoliosis	Caudal fin abnormalities
WT vs asmt1$^{+/-}$	ns	ns	ns
WT vs asmt$^{-/-}$	P < 0.001	P < 0.001	ns
WT vs asmt2$^{+/-}$	ns	ns	ns
WT vs asmt2$^{-/-}$	P < 0.01	ns	P < 0.05
asmt1$^{+/-}$ vs asmt1$^{-/-}$	P < 0.001	P < 0.001	ns
asmt1$^{+/-}$ vs asmt2$^{+/-}$	ns	ns	ns
asmt1$^{+/-}$ vs asmt2$^{-/-}$	P < 0.01	ns	ns
asmt1$^{-/-}$ vs asmt2$^{+/-}$	P < 0.001	P < 0.001	ns
asmt1$^{-/-}$ vs asmt2$^{-/-}$	P < 0.01	P < 0.001	ns
asmt2$^{+/-}$ vs asmt2$^{-/-}$	P < 0.001	ns	P < 0.05
